# Phase retrieval by coherent modulation imaging

**DOI:** 10.1038/ncomms13367

**Published:** 2016-11-18

**Authors:** Fucai Zhang, Bo Chen, Graeme R. Morrison, Joan Vila-Comamala, Manuel Guizar-Sicairos, Ian K. Robinson

**Affiliations:** 1 London Centre for Nanotechnology, University College London, London WC1E 6BT, UK; 2 Research Complex at Harwell, Harwell Campus, Didcot OX11 0FA, UK; 3 Department of Electrical and Electronic Engineering, Southern University of Science and Technology, Shenzhen 518055, China; 4 School of Materials Science and Engineering, Tongji University, Shanghai 201804, China; 5 Swiss Light Source, Paul Scherrer Institut, CH-5232 Villigen PSI, Switzerland; 6 Condensed Matter Physics and Materials Department, Brookhaven National Lab, Upton, New York 11973, USA

## Abstract

Phase retrieval is a long-standing problem in imaging when only the intensity of the wavefield can be recorded. Coherent diffraction imaging is a lensless technique that uses iterative algorithms to recover amplitude and phase contrast images from diffraction intensity data. For general samples, phase retrieval from a single-diffraction pattern has been an algorithmic and experimental challenge. Here we report a method of phase retrieval that uses a known modulation of the sample exit wave. This coherent modulation imaging method removes inherent ambiguities of coherent diffraction imaging and uses a reliable, rapidly converging iterative algorithm involving three planes. It works for extended samples, does not require tight support for convergence and relaxes dynamic range requirements on the detector. Coherent modulation imaging provides a robust method for imaging in materials and biological science, while its single-shot capability will benefit the investigation of dynamical processes with pulsed sources, such as X-ray free-electron lasers.

## Abstract (web summary)

Robust coherent diffractive imaging generally requires many exposures that may damage samples. Here, the authors develop a single-shot X-ray imaging method applicable to general samples for materials and biological sciences, also enabling imaging of dynamic processes, using a pulsed X-ray laser.

## Graphical abstract

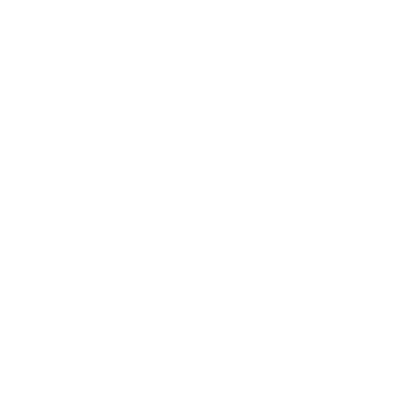

Phase retrieval arises in fields such as electron microscopy^1^, crystallography^2^ and astronomy^3^, and is a core aspect of coherent diffraction imaging (CDI)^1^,^4^,^5^,^6^. Iterative projection algorithms^7^,^8^ commonly used in CDI search for a solution by iteratively using projections and constraints in two separated planes. Since the first demonstration of CDI with X-rays^4^, the high brightness available at third-generation synchrotrons and X-ray free-electron lasers (XFELs)^9^,^10^ along with the development of more sophisticated algorithms and experimental set-ups^11^,^12^,^13^,^14^, has led to impressive results in materials and biological science, such as imaging of nano-particles^15^, cells^16^ and viruses^17^.

The current challenges for CDI include the high sensitivity of reconstruction convergence to noise or missing experimental data^17^,^18^,^19^, the limited ability to retrieve images for extended objects or complex-valued samples from single measurements^20^, and the need to collect data with a high dynamic range^17^,^19^,^21^. Various modifications to the illumination^22^,^23^,^24^ have been proposed to address those issues for which the object appears as a perturbation on the illumination. A substantial *a priori* knowledge of the exit wave reduces the possibility that the algorithm becomes stagnated. Modifications to the sample exit wave have also been suggested^25^,^26^,^27^ in which multiple measurements have been a key requirement for the convergence of reconstruction. We note that single-shot imaging schemes that use aperture arrays^28^,^29^ or structured light to illuminate a sample^24^ have recently been proposed, but so far only simulations and visible-light demonstrations have been reported.

The phase problem in CDI is usually formulated as recovering an image from its Fourier-magnitude data. In this paper we show that breaking the Fourier transform relation by inserting a wavefront modulator between the sample and the detector can greatly help to solve the phase problem with a single measurement. This coherent modulation imaging (CMI) method was initially demonstrated with visible light^30^. Here we extend its application to the X-ray regime using a substantially improved algorithm and show that CMI addresses many of the challenges that affect conventional CDI.

## Results

### Description of the experiment

**Figure 1:**
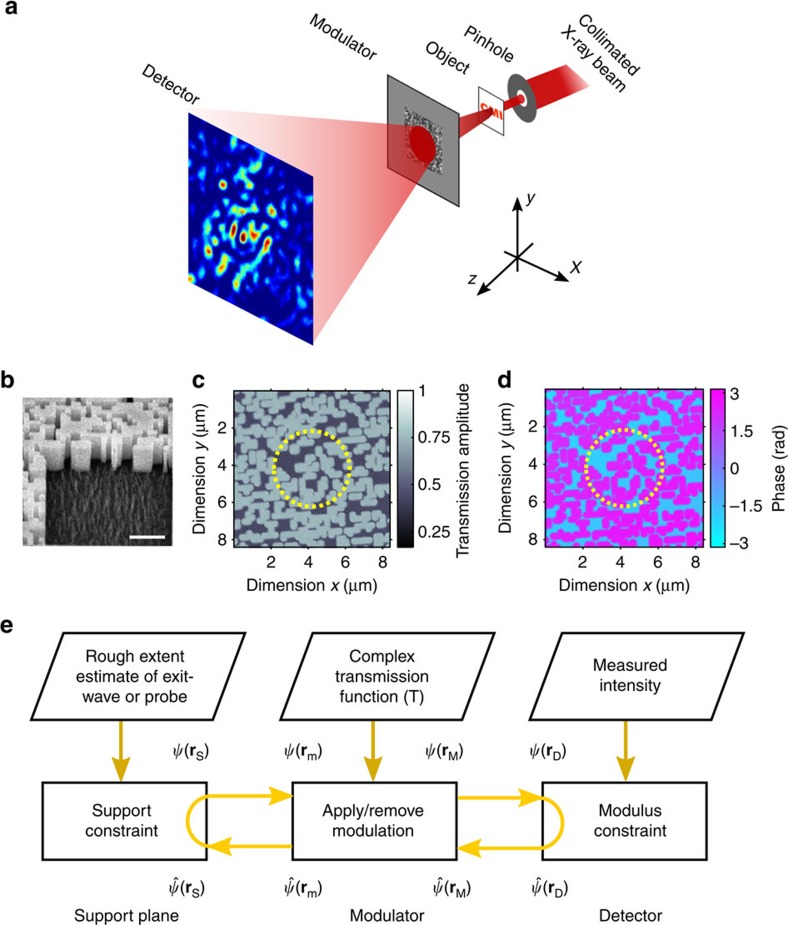
Key aspects of CMI. (**a**) Schematic of the CMI set-up. (**b**) Tilted scanning electron microscope view of the fabricated modulator; scale bar, 2 μm. (**c**,**d**) Modulator amplitude (**c**) and phase (**d**) measured by ptychography; the dashed yellow circles illustrate the relative size of the illumination probe. (**e**) Flowchart of the three-plane phase retrieval algorithm; the arrows indicate wave-propagation steps; the top row shows the input information on each plane.

Our CMI experiments were conducted at the cSAXS (coherent small-angle X-ray scattering) beamline of the Swiss Light Source. The experimental set-up is illustrated in Fig. 1a. Coherent X-rays of energy 6.2 keV passed through a 2.2 μm diameter pinhole, forming an illumination probe with a photon flux of 3.6 × 10^6^ photons per second on an object located 1 mm downstream. The X-rays transmitted by the object then passed through a wavefront modulator 2.5 mm further downstream before propagating 7.2 m to a Pilatus 2M detector with square pixels with a side length of 172 μm (ref. 31). The central 512 × 512 pixels of the recorded pattern were used for reconstruction. The effective image pixel size at the planes of the modulator and the object was 16.4 nm.

Placing a wavefront modulator downstream of the sample is essential in this method. In our experiments, the modulator consisted of randomly distributed tungsten pillars with a nominal width and spacing of 200 nm. A tilted view of the modulator structure obtained using a scanning electron microscope is shown in Fig. 1b. The transmission of the modulator was characterized using ptychography^32^,^33^,^34^ before measurements of the sample using CMI (Methods). The modulator transmission functions are shown in Fig. 1c,d, and these were used as *a priori* information in the CMI phase retrieval algorithm. In our experiments, about 70% of the X-rays were transmitted through the phase plate modulator and used for reconstruction. The fluence on the modulator was calculated to be 0.24 J cm^−2^, which is below the damage threshold of 0.5 J cm^−2^ found for Fresnel zone plates made of tungsten and is well below the threshold of 59 J cm^−2^ for Fresnel zone plates made of diamond when exposed to free-electron laser pulses^35^. No beam-induced damage to the modulator was observed during the course of the experiments.

A flowchart of the CMI phase retrieval algorithm is shown in Fig. 1e. The support plane is one where the wavefield is known to have non-zero values within a finite region. It may correspond to the sample plane, but this is not a general requirement. Accurate knowledge of the axial separation between the sample and the modulator is not critical. Numerical wave propagation after reconstruction can be performed to bring the retrieved sample exit wave to focus when necessary. Reconstruction in CMI is performed by iteratively propagating a wavefield estimate between the support and detector planes, via the modulator. The applied constraints and the necessary inputs in each plane are indicated in Fig. 1e. The steps of modulation and wave propagation between the modulator and the support plane yield a rapid algorithmic convergence due to the removal of the twin image and spatial shift ambiguities whose competition with the correct solution is a potential cause of stagnation in conventional algorithms that use two planes^36^,^37^. Ambiguity removal in CMI is discussed in more detail in the Supplementary Note 1 and Supplementary Fig. 1. Details of the newly developed three-plane phase retrieval algorithm are given in the Methods section.

### Single-shot reconstructions with a relaxed support

**Figure 2:**
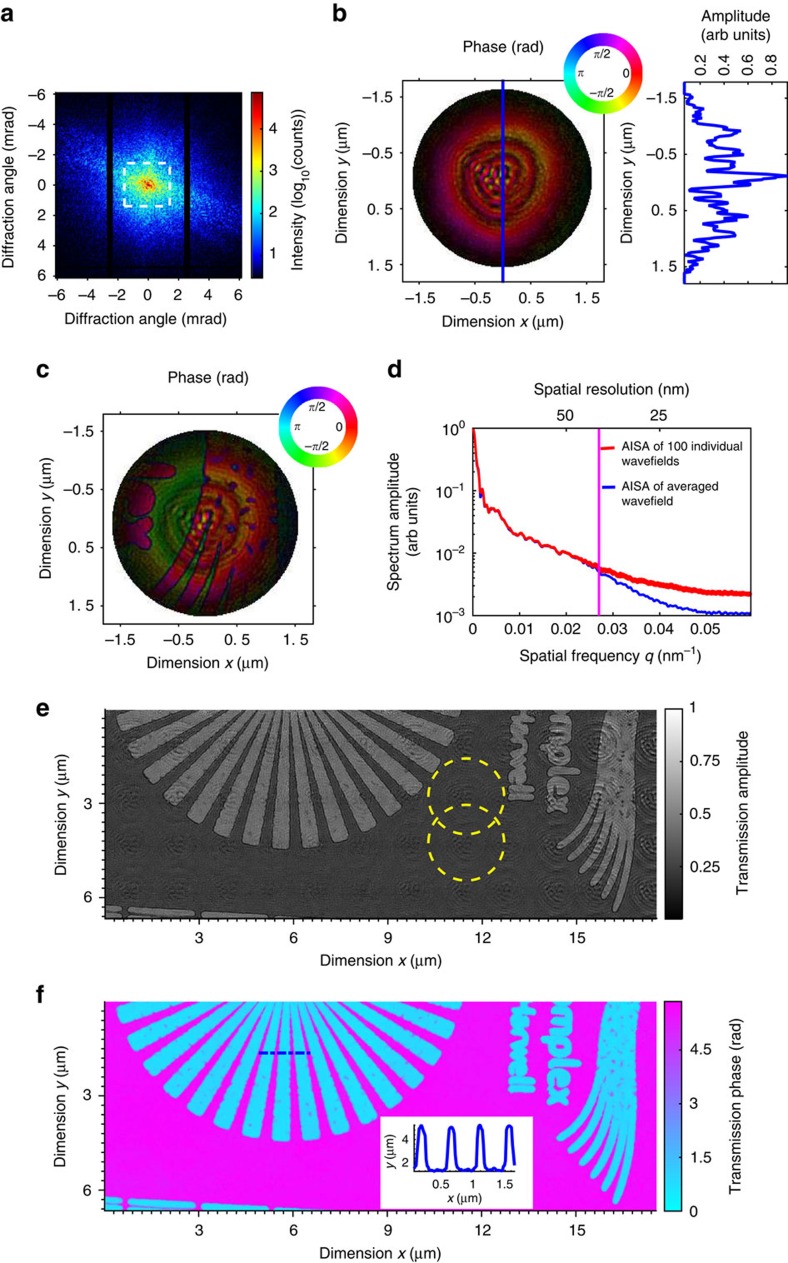
Imaging an extended test pattern by CMI. (**a**) An example of a recorded diffraction pattern, shown on a logarithmic scale; the dashed square indicates the extent of the data used for the missing data simulation; the vertical black strips are gaps between the detector modules. (**b**) The reconstructed illumination wavefield with amplitude displayed as brightness and phase as hue according to the inset phase ring. The circular rim of the reconstruction indicates the support. The amplitude profile on the right side is taken along the central vertical line and shows slowly tapering edges. (**c**) A reconstruction from data in **a** of the wavefield on the exit surface of a part of the sample. The wavefield is the product of the complex probe amplitude and the sample transmittance. (**d**) Plots of the azimuthally integrated spectrum amplitude (AISA) from 100 retrieved wavefields with different starts (red curves) and of the averaged wavefield (blue curve). The vertical magenta line indicates the point of 5% deviation between blue and red curves, which provides a resolution estimate of 37 nm. (**e**,**f**) Stitched amplitude and phase images from a 12 × 5 array of overlapping single-shot reconstructions as in **c**. The two yellow circles illustrate the size and overlap for neighbouring positions of the illumination probe. The inset in **f** is a phase line profile plot along the blue dashed line.

**Figure 3:**
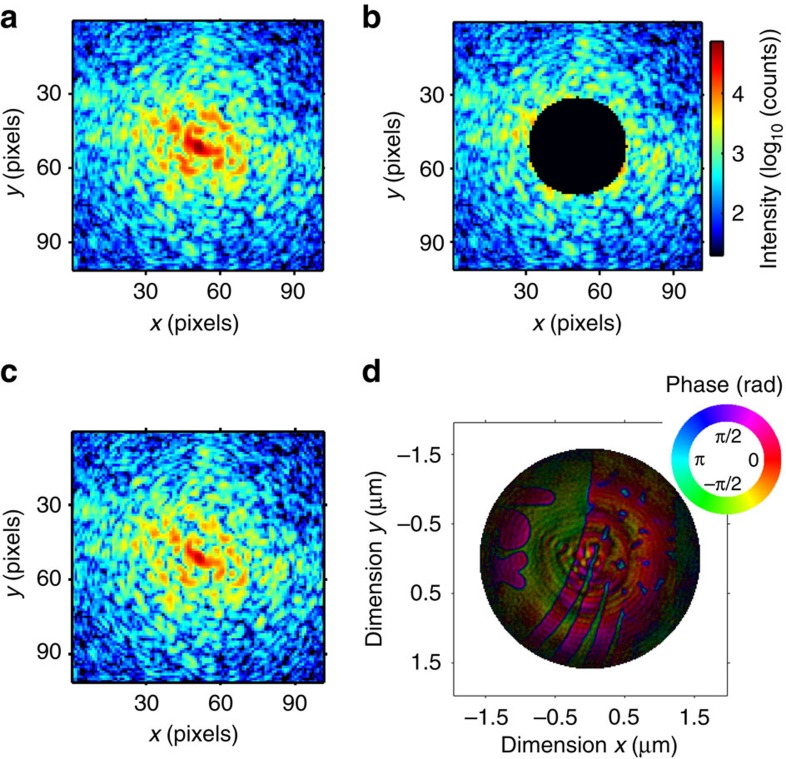
Robustness of CMI to missing data. (**a**) The central 100 × 100-pixel region of a recorded intensity pattern. (**b**) The same pattern as in **a** but with the central 40-pixel diameter disc masked out. (**c**) Diffraction pattern recovered by the CMI algorithm. The colour bar in **b** also applies to **a** and **c**. (**d**) Reconstruction from the data with missing centre as shown in **b**, with the amplitude and phase displayed as image brightness and image hue with colour coding of the inset colour ring.

An example of CMI data recorded with a 3 s exposure time is shown on a logarithmic scale in Fig. 2a. The dashed square indicates the extent of the diffraction data used for the simulation of missing data shown in Fig. 3a–c. The requirement for high dynamic range detection and the consequent need for a beam stop are reduced in CMI (Supplementary Fig. 2). The illumination wavefield shown in Fig. 2b was retrieved from a single-shot CMI measurement after 150 computational iterations with the loose support of a 3.2 μm-diameter disk. The amplitude is displayed as image brightness and the phase as image hue. The amplitude profile on the right side of Fig. 2b is taken along the central vertical line. It shows that the illumination oscillates on either side of the central maximum and has slowly tapered edges. The overall probe width is around 2.4 μm, measured to where the edge taper falls to half the value of the central maximum. The support requirement on the object exit wave can be significantly relaxed in CMI and this allows robust reconstruction even in the case of a tapered wavefield that would pose difficulties for conventional phase retrieval algorithms^38^. In our experiment, the modulator centre was approximately aligned with the illumination beam. A loose support centrally located around the sample exit wavefield was found sufficient to recover an image from the recorded data. In practice, the lateral object position relative to the modulator only needs to be known roughly to provide an initial estimate of the support position. The exact lateral position of the object wave relative to the modulator can be retrieved automatically by the phasing algorithm as long as a significant part of the wavefield falls inside the specified support. This is shown in Supplementary Fig. 3b, where a part of the wave can still be reconstructed when the chosen support is laterally displaced. The resulting reconstruction gives a clear indication of where to reposition the support. In our experiments, the detector was in the far field of the modulator so the lateral position of the modulator relative to the detector did not affect the behaviour of the reconstruction algorithm. We have found the new CMI algorithm very robust to changes of the modulator's lateral position.

The CMI reconstruction of part of a test pattern nanofabricated in a 1.5 μm thick tungsten film by electron beam lithography and reactive ion etching is shown in Fig. 2c. The amplitude variations within the probe cause some parts of the sample to be illuminated with fewer photons, and thus affect the reconstruction quality of those areas. A flat-top illumination would therefore be preferable. A 3.2 μm diameter disk defined a loose support for the exit wavefield, and during the single-shot reconstruction the sample features started to appear after only a few tens of iterations from a random initial guess (Supplementary Video 1). A reconstruction using the looser support of a 3.7 μm diameter disk is shown in Supplementary Fig. 3a and is of comparable quality to Fig. 2c. A sequence of single-shot reconstructions covering a larger region of the test pattern is shown in Supplementary Video 2, where the phase of the illumination wavefield has been subtracted to reveal the phase change induced by the object.

We estimated the reconstruction resolution from the range of spatial frequencies of the retrieved wavefields that were stable for different initial guesses. In CMI the recorded data are related to the object via a modulator whose transmission function might not be known accurately. Resolution metrics that compare the computed estimate directly with the recorded data, such as the phase retrieval transfer function^21^,^39^, are not readily suitable. The red curves in Fig. 2d show the azimuthally integrated spectral amplitude (AISA) for 100 individually retrieved wavefields with different random starts. These wavefields were also averaged after their arbitrary phase offsets have been removed, and the blue curve shows its corresponding azimuthally integrated spectral amplitude. The blue curve deviates from the red ones at a spatial frequency, where the data noise starts to dominate the fine structure in the image. A 5% deviation indicated by the vertical line gives a resolution estimate of 37 nm.

In our experiments, the allowed wave extent on the modulator was 4.2 μm to fulfil the Nyquist sampling condition of the diffraction intensity in each dimension. This sets a limit on the sample area that can be imaged from a single measurement. In our experiments, however, a small fraction of the light was spread outside this area due to wave propagation effects from the pinhole to the modulator plane. To reduce the possible artefacts arising from this spreading, we performed reconstructions with a larger object window and applied the modulus constraint with suitable down-sampling and up-sampling operators (Methods).

### Extension of the field of view

CMI can retrieve the sample exit wave from a single measurement or the illumination probe when no sample is present. If multiple measurements are taken from overlapping regions of a thin object, the overlap update approach used in ptychography^34^ can be used to separate the object from the illumination and to stitch together an image of a larger region of the object. The absorption and phase images stitched from a 12 × 5 array of single-shot reconstructions of the test sample, covering an area of 17.5 × 6.65 μm^2^, are shown in Fig. 2e,f. The 40% linear overlap between neighbouring illuminated regions illustrated by yellow circles in Fig. 2e was chosen to ensure full coverage of the sample area when stitching the single-shot reconstructions together. The object translations were determined retrospectively by aligning the single-shot reconstructions^40^, which avoided the positioning errors of the stepper motors. Some artefacts bearing the amplitude profile of the illumination probe can be seen, and these are due to small changes in the probe between each single-shot measurement. When the probe function is stable over a number of shots, a measurement of the probe before the measurements on the sample can be used to reduce such artefacts by dividing out the illumination from the exit wave to provide the sample transmission, provided that the probe amplitude is well above the noise level.

### Tolerance to missing data

In CDI, the diffraction data missing from a measurement will result in unconstrained spatial modes in the phase retrieval that hinder convergence^17^,^18^,^21^. The robustness of CMI to missing data was demonstrated by reconstructing measured diffraction data with its central region of 40 pixels in diameter being deliberately masked out, as shown in Fig. 3b. The speckles omitted from the centre were well retrieved as shown in Fig. 3c and are comparable to the recorded data in Fig. 3a. The quality of the retrieved image in Fig. 3d is comparable to that in Fig. 2c.

### Reconstructions of a zone plate

**Figure 4:**
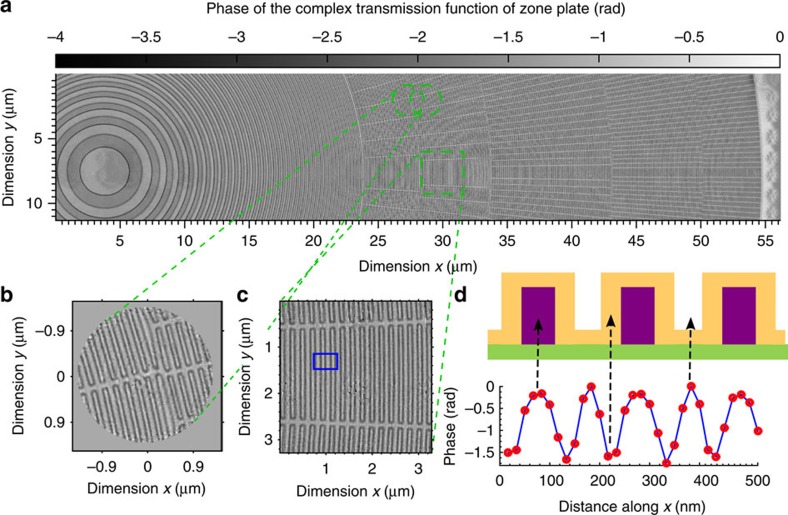
Imaging of a zone-doubled Fresnel zone plate by CMI. (**a**) Phase delay image stitched together from an array of 39 × 8 overlapping CMI reconstructions. The green yellow circles illustrate the overlap of two adjacent areas illuminated by the probe. (**b**) Enlarged single-shot CMI reconstruction of the circled region. (**c**) Enlarged image of the boxed region of **a**. (**d**) Top: a cross-section of the design: green, Si_3_N_4_ membrane; yellow, iridium coating; magenta, hydrogen silsesquioxane resist; the aspect ratio is not to scale. Bottom: red dots are phases of reconstructed pixels averaged vertically within the box in **c**; blue line is a linear fit; the nominal 30 nm-thick iridium coating is clearly revealed in the line profile.

In addition to the strongly scattering test sample, we performed CMI on a weaker sample of a zone-doubled Fresnel zone plate with buried structures^41^. The zone plate was made of hydrogen silsesquioxane resist on a Si_3_N_4_ membrane, patterned by electron beam lithography and coated with a uniform and conformal thin film of iridium by atomic layer deposition. The zone plate has a diameter of 100 μm and an outermost zone width of 25 nm. A stitched phase image from a 39 × 8 array of single-shot reconstructions of the zone plate sample, covering an area of 56 × 11 μm^2^, is shown in Fig. 4a (the absorption image is shown in Supplementary Fig. 4). As one example, the single-shot reconstruction of the left circled region obtained using the loose support of a 3.2 μm disc is shown in Fig. 4b. Figure 4c is a close-up view of the boxed area in Fig. 4a. The iridium coating with a nominal thickness of 30 nm is clearly revealed on the steep sidewall of the photoresist, as shown in Fig. 4d. The buried hydrogen silsesquioxane resist pillars shown in the cross-section in Fig. 4d cannot be visualized by surface-sensitive techniques such as scanning electron microscopy, demonstrating one advantage of transmission X-ray imaging.

## Discussion

We have shown a practical solution to the phase problem with a single exposure of the sample that applies to any form of coherent radiation and to samples of different diffracting strengths and physical sizes. Using a wavefront modulator we break the Fourier transform relationship between the object and the measured data. The method overcomes several challenges with conventional single-shot CDI. The CMI phase retrieval algorithm converges rapidly with only a loose support. In addition, we show the method can robustly recover significant areas of missing diffraction data, which can occur experimentally when beam stops are used or when the detector has a segmented structure with gaps between the segments. In addition, the use of a wavefront modulator can substantially reduce the intensity of the central beam and spread the photons more evenly over the detector, thus reducing the dynamic range requirement on the detector and consequently the need for a beam stop. The reduction of the data dynamic range is especially important for experiments on XFELs, where non-counting detectors of limited dynamic range, such as charge-coupled devices, are commonly used. Diffractive optics made from diamond and tungsten have been successfully tested and found to survive XFEL pulses^35^,^42^, and these materials could be used to form a radiation-resistant wavefront modulator for XFEL CMI applications.

The single-shot capability of CMI can potentially become an important asset in circumventing the resolution limits imposed due to radiation damage^43^,^44^ in X-ray microscopy, in the same way that the imaging of single biomolecules was first proposed^45^ and later demonstrated^46^ using XFELs. The development of single-shot diffraction imaging techniques in combination with the ultrashort pulses of highly brilliant XFEL sources is a promising method to record the diffraction pattern of biological samples before the radiation damage reveals itself. On one hand, it is complementary to current efforts to add cryogenic capabilities to existing ptychographic coherent diffraction imaging^47^ and X-ray microscopy systems^48^,^49^ to preserve the state of radiation-sensitive samples during data acquisition. On the other hand, the single-shot capability offers the possibility to study ultrafast dynamical processes with a temporal resolution limit only determined by the pulse structure of the light source and the rate at which the detector can acquire successive diffraction patterns.

## Methods

### Design considerations for the modulator

There are two considerations in the design of the transmission function of the modulator: to reduce the data dynamic range and to resolve ambiguities during phase retrieval. The exact form of modulator function is not critical and it is not restricted to a particular form such as to resemble a Fresnel zone plate. The resolution of the reconstructed image is not constrained by the feature size of the modulator. For ease of fabrication by electron beam lithography, a metal film with a binary pattern of open areas and phase-shifting areas was used in our experiment. The density of phase-shifting areas was designed to minimize the intensity of the zero-order component and hence to reduce the data's dynamic range. The angular spectrum of the light scattered by the modulator should fall within the angular acceptance of the detector. For the removal of ambiguities, a broader angular spectrum and a larger variation of the modulation function would discriminate the real solution from the ambiguous ones more effectively (Supplementary Fig. 1). In our simulation and experiments with various samples, the convergence rate of a reconstruction and the quality of the resulting images showed little dependence on the wavefront (sample) structures when the modulator phase varies more strongly than the wavefield that was retrieved. Our set-up and the design of the modulator have been found to work for general samples.

In our experiment, the modulator consisted of a silicon nitride support membrane coated with a tungsten film into which a randomly distributed pattern of 200 nm wide spaces was etched using electron beam lithography. Figure 1b shows an scanning electron microscope image of the modulator taken at a tilted angle to reveal the fabrication quality. The design thickness of the tungsten coating was 1.26 μm to induce a *π* radians phase shift at an X-ray energy of 6.2 keV. The pillar density was chosen to ensure their contribution to the zero-order diffracted amplitude was comparable to the contribution from the open spaces. The relative phase difference of these contributions was designed to be *π* radians, resulting in destructive interference that reduces the strength of the zero-order beam. In this way, the need for a beam stop was reduced and the dynamic range requirement of the detector was relaxed.

### Characterization of the modulator

The phase and amplitude of the modulator transmission function were used as *a priori* information in our calculation and were measured by ptychography^32^,^33^,^34^ before the CMI single-shot measurements. A total of 177 diffraction patterns over an area of 12 μm × 12 μm of the modulator were recorded as the modulator was scanned across the X-ray beam using a two-axis piezo stage. The scan positions lay at the vertices of concentric polygons, starting with a pentagon. There was a radial increment of 0.8 μm and the number of vertices increased by five between successive rings, with an exposure time of 3 s at each scan point. The extended ptychographical iterative engine algorithm with position correction^50^ was run on the recorded data for 120 iterations, yielding the results shown in Fig. 1c,d. The phase shift due to the tungsten pillars was measured to be 4.1 radians, 33% larger than the design value.

### Reconstruction algorithm

The algorithm used here is an improved version of the one described in ref. 30. A new de-modulating approach has been used, which is suitable for a modulator with a strong absorption variation. The raised-power magnitude constraint^30^ has been replaced by the approach described below in step (4) to prevent the algorithm from being trapped in local minima. The magnitude constraint in the detector plane has been revised to include up-sampling and down-sampling at the detector plane as described below in step (5). The results presented in this paper were only possible with these improvements to the algorithm.

The wave estimates at the support, modulator and detector planes are specified by their coordinate subscript S, m or M and D, respectively. Normally we start with a random initial guess at the support plane and proceed as follows for the *j*th iteration,

Apply the support constraint to drive towards zero all values outside the support



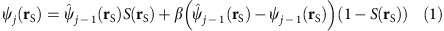

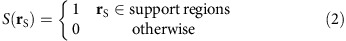
 where *S*(**r**_S_) denotes the support; *ψ*_*j*−1_(**r**_S_) is the previous estimate; and 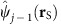 is the estimate after revising by the measured data in the (*j*−1)th iteration, as shown in Fig. 1e. The estimate outside the support is gradually driven towards zero at a rate controlled by the constant *β*; throughout the results reported here *β*=0.5 was used.

Propagate *ψ*_*j*_(**r**_S_) to the modulator, which yields *ψ*_*j*_(**r**_m_). For the results presented in this paper, the angular spectrum method was used for propagation between the support plane and the modulator.

Apply modulation


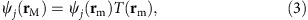


where *T* (**r**_m_) is the complex transmission function of the modulator. The subscripts m and M represent planes directly upstream or downstream of the modulator.

Generate the estimate at the detector plane according to


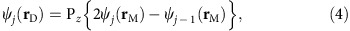


where P_*z*_{·} represents the wave propagator for a distance *z* from the modulator to the detector and was a Fourier transform in our case. Every *N* iterations, where *N*=40 in the paper, *ψ*_*j*−1_(**r**_M_) is set to equal *ψ*_*j*_(**r**_M_), which appeared to help to avoid stagnation.

Apply the magnitude constraint

To accommodate some energy outside of the object space area defined by the Nyquist sampling condition at the detector, we use estimates of the wavefield with a larger array size than the recorded diffraction pattern. A larger array size in the calculation accounts for some degree of under-sampling in the diffraction data and helps reduce artefacts. This has been found beneficial in improving the image quality of our results.

After calculating the diffracted wave *ψ*_*j*_(**r**_D_) at the detector, we down-sampled its amplitude to match the sampling interval of the diffraction pattern. First, the amplitude of the calculated wave *ψ*_*j*_(**r**_D_) is convolved with a detector pixel form function and then down-sampled to the array size of the recorded data, yielding |*ψ*_*j*_(**r**_D_)|_ds_. A constant function of 2 × 2-pixel wide was used to approximate the detector pixel form function for our results. Next, we calculated the weighting function *β*_ds_(**r**_D_) of the measured data with respect to the down-sampled amplitude, according to


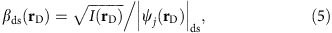


and then up-sampled the weighting function, here using nearest-neighbour interpolation, yielding *β* (**r**_D_); where *I*(**r**_D_) is the measured intensity in the detector plane. Finally, the revised wavefield at the detector is calculated as


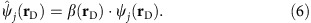


Back-propagate the revised wave at the detector to the modulator plane to form a revised exit wave downstream of the modulator


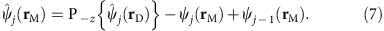


Update the wavefield upstream of the modulator by


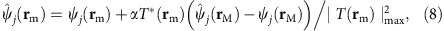


where *α* is a constant. For the results in this paper *α*=1.

Back-propagate 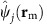 to the support plane, yielding the revised wave field 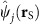.

Steps 1–8 are repeated until a termination condition is met.

The algorithm for wavefield propagation in step (2) between the support plane and the modulator, and in step (4) between the modulator and detector, should be adapted to the experiment geometry. For near-field propagation, the angular spectrum method is used; the Fresnel propagator is more suitable for the case of intermediate-range propagation^51^.

### Data availability

All the relevant data are available from the authors on request.

## Additional information

**How to cite this article:** Zhang, F. *et al*. Phase retrieval by coherent modulation imaging. *Nat. Commun.* **7,** 13367 doi: 10.1038/ncomms13367 (2016).

**Publisher's note:** Springer Nature remains neutral with regard to jurisdictional claims in published maps and institutional affiliations.

## Supplementary Material

Supplementary Information Supplementary Figures 1-4, Supplementary Note 1 and Supplementary References.

Supplementary Movie 1 The video shows the CMI reconstruction process with iterations. Progress of single-shot coherent modulation imaging reconstruction versus iterations.

Supplementary Movie 2 The video shows multiple single-shot CMI reconstructions of an extended sample. Single-shot coherent modulation imaging over an extended sample.
