# Supplementary material for: Phase retrieval by coherent modulation imaging
Source: Nat Commun. 2016 Nov 18;7:13367. doi: 10.1038/ncomms13367 (PMC5120206; doi:10.1038/ncomms13367)
Supplement: Supplementary Information — Supplementary Figures 1-4, Supplementary Note 1 and Supplementary References. [file ncomms13367-s1.pdf]

Supplementary Figures

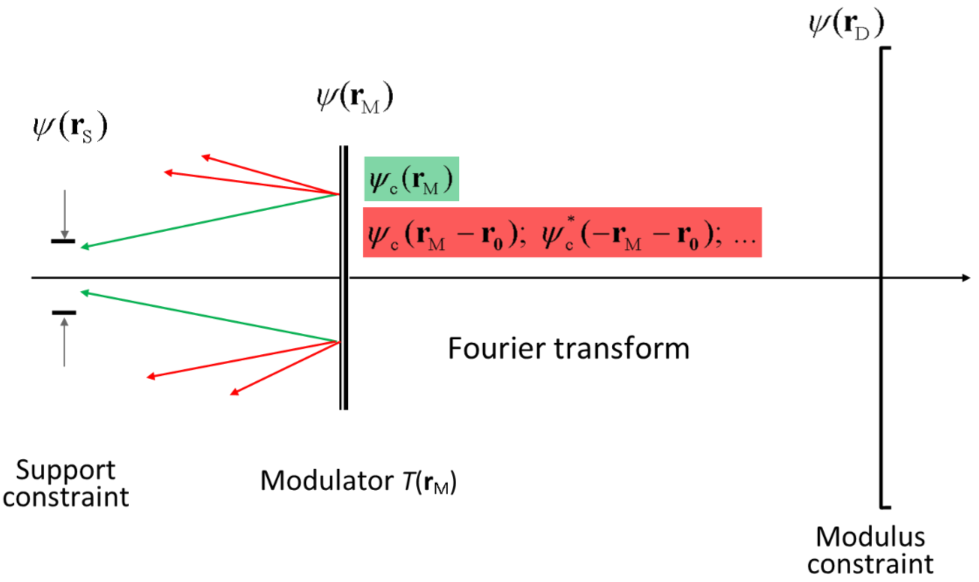

Supplementary Figure 1 Illustration of the removal of ambiguous solutions in the iterative calculation of coherent modulation imaging. The items coloured in green and red indicate the correct and the ambiguous wave components respectively. Ambiguous wave components conflict with the support constraint and are gradually eliminated as the iteration progresses.

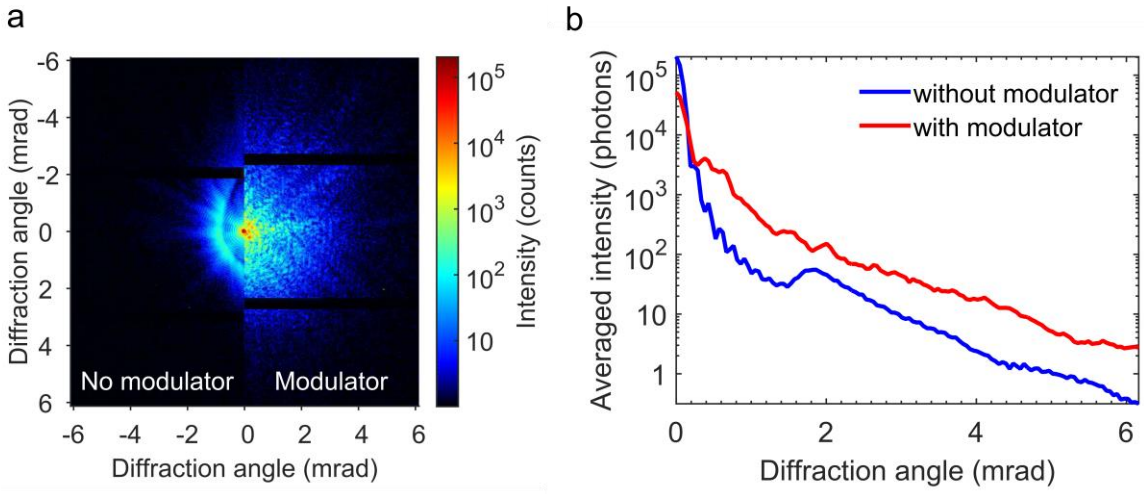

Supplementary Figure 2 Reduction of data dynamic range in coherent modulation imaging. a, Recorded data on the detector for the illumination probe with a modulator (right) and without modulator (left), displayed on a logarithmic scale. **b,** Comparison of the azimuthally averaged intensity distribution versus diffraction angle. With a modulator, the intensity of the central spot was reduced by a factor of 4; the polar-averaged intensity was enhanced by a factor of 8 at a diffraction angle of 1.4 mrad, and a factor of 5 at an angle of 5 mrad. The still dominant residual central beam intensity is due in part to the fact that the modulator was fabricated about 33% thicker than the design value.

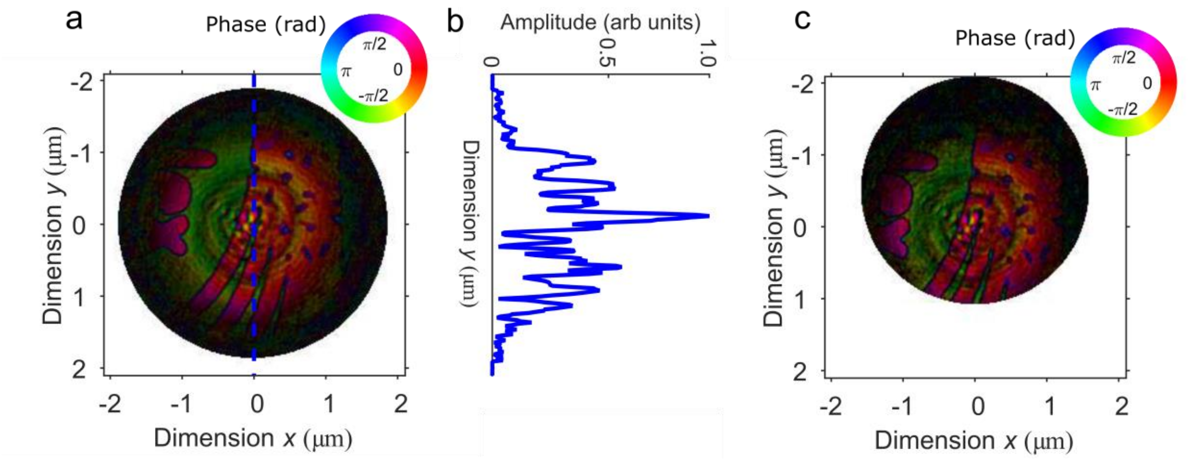

Supplementary Figure 3 Robustness of coherent modulation imaging to a loose or misplaced support. a, Reconstructed exit-wave of a test sample with a 3.7 μm support. **b,** Vertical line cut through the image centre in (**a**), showing the slowly tapered edges of the object exit-wavefield. **c,** Reconstructed exit-wave with a 3.2 μm support misplaced by 0.5 μm (30 pixels). In (**a**) and (**c**), the amplitude is displayed as the image brightness and the phase is displayed as image hue over the range of [-π, π] using colour encoding of the inset colour ring. The image quality of (**a**) is compatible to result from using a centred 3.2 μm support. Although part of the retrieved wave field in (**c**) was cut off by the support, the reconstruction still shows distinct object features. This partial reconstruction can be used to refine the initial estimate of the support position.

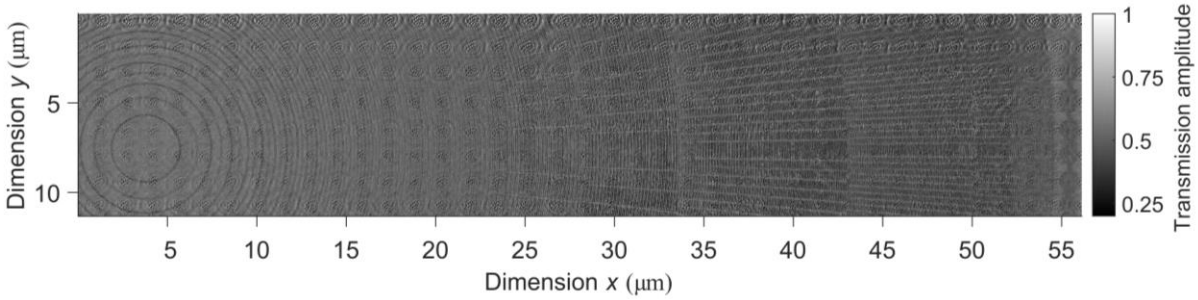

Supplementary Figure 4 Absorption image of zone-doubled Fresnel zone plate. Stitched from 39×8 single-shot reconstructions.

Supplementary Notes

Supplementary Note 1 Removal of phase retrieval ambiguity in Coherent Modulation Imaging (CMI). In the conventional implementation of coherent diffraction imaging (CDI), the samples are illuminated by a planar wavefront and their diffraction intensity patterns are recorded in the far field. As a result, the object wave and the diffracted wave at the detector plate are related by a Fourier transform. When a finite support is used as the object constraint, the associated phase problem has non-unique solutions[1-3]. For example, if $\psi(\mathbf{r})$ is the solution to the phase problem, the Hermite conjugate $\psi^*(-\mathbf{r})$ (the twin image) and the spatial shift $\psi(\mathbf{r}-\mathbf{r_0})$ are also solutions if they can satisfy the support constraint, where $\mathbf{r}$ is a coordinate vector in the sample plane and $\mathbf{r_0}$ is a constant offset. Although any these functions could be regarded as a valid solution, a combination of them can appear in the intermediate estimates during the iterative calculation and cause the phase retrieval algorithm to converge slowly or even fail.

One key property of CMI is the ability to eliminate the twin-image and the spatial-shift ambiguities. Supplementary Figure 1 visually illustrates how such ambiguities are removed in the iterative calculations. Note the correct wave downstream of the modulator, $\psi_\mathrm{c}(\mathbf{r}_\mathrm{M})$, in colour green and the other ambiguous solutions in red in the Supplementary Figure 1. In the reconstruction, these ambiguous wave components coloured in red would initially occur after applying the modulus constraint at the detector. After the operations of demodulation and back-propagation to the support plane, only the correct wave component can fulfil the support constraint. Components corresponding to the ambiguous solutions would spread outside the support and would be gradually removed by the support constraint as the iteration progresses. A strong modulation and a large separation between the modulator and the support plane will result in a larger portion of the ambiguous components being removed by the support constraint and thus increase the efficiency of ambiguity elimination.

Supplementary References

1. Fienup, J. R. & Wackerman, C. C. Phase-retrieval stagnation problems and solutions. *J. Opt. Soc. Am. A* **3**, 1897-1907 (1986).
2. Seldin, J. H. & Fienup, J. R. Numerical investigation of the uniqueness of phase retrieval. *J. Opt. Soc. Am. A* **7**, 412-427 (1990).
3. Guizar-Sicairos, M. & Fienup J. R. Understanding the twin-image problem in phase retrieval. *J. Opt. Soc. Am. A* **29**, 2367-2375 (2012).
